# Nanomaterials: breaking the bottleneck of breast cancer drug resistance

**DOI:** 10.3389/fimmu.2024.1492546

**Published:** 2024-11-13

**Authors:** Chao Guan, Yahao Han, Zhenzheng Ling, Xiang Meng, Baolin Zhang, Wanwei Dong, Di Zhang, Keyan Chen

**Affiliations:** ^1^ The First Clinical College of China Medical University, Shenyang, Liaoning, China; ^2^ Laboratory Animal Science of China Medical University, Shenyang, Liaoning, China; ^3^ The Fourth Clinical College of China Medical University, Shenyang, Liaoning, China; ^4^ Department of Cardiology, Shengjing Hospital of China Medical University, Shenyang, Liaoning, China

**Keywords:** breast cancer, nanomaterials, anticancer drugs, drug resistance, targeted therapy

## Abstract

Drug resistance poses a significant challenge in the treatment of breast cancer. In recent years, a variety of nanomaterials have been discovered and synthesized that can selectively target tumor cells and play a crucial role in the advancement of breast cancer therapies. As our understanding of tumor heterogeneity deepens, the emerging potential of nanomaterials in addressing drug resistance has garnered considerable attention. These materials not only selectively target tumor cells but also possess unique properties that make them promising options for cancer treatment, including low toxicity, excellent biocompatibility, ease of preparation, the ability to carry antitumor drugs, and customizable surface functions. In this review, we will comprehensively summarize two key developments in breast cancer treatment: the application of antitumor drugs and nanomaterials. We will explore the mechanisms by which nanomaterials improve drug resistance in breast cancer, targeted nanotherapy strategies to mitigate this resistance, and recent research advancements in anticancer nanomaterials. This overview aims to highlight the significant role of nanomaterials in breast cancer treatment and provide a theoretical framework for identifying optimal treatment strategies in the future.

## 1 Introduction

Breast cancer has the highest mortality rate among female cancers in many countries and regions, surpassing lung cancer to become the most commonly diagnosed cancer (1–3). According to the American Cancer Society (1), there will be approximately 300,590 new cases of breast cancer in 2023, resulting in an estimated 43,700 deaths.

Currently, the primary treatments for breast cancer include surgery, along with radiotherapy, chemotherapy, hormone therapy, and other methods (4). Chemotherapy is a widely used approach; however, its lack of selectivity for tumor tissues presents significant challenges (5–7). These challenges include systemic toxicity and the accumulation of chemotherapy drugs at tumor sites, which can lead to tumor resistance (8).

Breast cancer cells can develop drug resistance through a variety of complex cellular and molecular mechanisms. These mechanisms primarily include (1) drug efflux and inactivation: tumor cells may diminish drug effectiveness by increasing drug efflux and decreasing intracellular drug concentrations (9). (2) Enhanced DNA damage repair: to resist DNA damage caused by chemotherapy drugs, tumor cells may enhance their DNA repair capabilities, which results in reduced drug efficacy (10). (3) Activation of bypass signals or pro-survival pathways: tumor cells can also manipulate apoptosis pathways, allowing them to evade chemotherapy-induced apoptosis and sustain their survival. (4) Epithelial-mesenchymal transition (EMT): this process can enhance tumor cell invasion and metastasis, potentially leading to increased resistance to chemotherapy drugs (11). (5) Stem-like properties: cancer stem cells may exhibit greater drug resistance, enabling them to sustain tumor growth and recurrence through their ability to self-renew and differentiate into other tumor cells (12). In summary, understanding and addressing the mechanisms of breast cancer drug resistance is crucial for improving treatment outcomes and enhancing patient survival rates.

In recent years, nanomaterials have shown significant potential in both the treatment and management of breast cancer (13). As research in nanotechnology has advanced, it has become evident that nanomaterials offer unique advantages in terms of biocompatibility, drug delivery (including drug release and localization) (14, 15), targeted therapy, antitumor effectiveness, and the ability to kill tumor cells *in vivo* (16). Additionally, the distinct properties of nanomaterials can influence breast cancer resistance mechanisms by controlling drug release (17, 18), thereby significantly alleviating drug resistance. Despite the variety of available nanomaterials, further study is needed to optimize their design, explore combined uses, and address toxicity concerns. Therefore, this review summarizes the latest advancements in the use of various nanomaterials for alleviating breast cancer resistance.

## 2 Current application status of anti-tumor drugs in breast cancer treatment

In the treatment of breast cancer, commonly used anti-tumor drugs contain chemotherapy agents such as cyclophosphamide and doxorubicin, as well as immunotherapy drugs like anti-PD-1/PD-L1 antibodies, including pembrolizumab and teslazumab. The selection of appropriate anti-tumor drugs is based on the molecular classification, clinical stage, and individual characteristics of each breast cancer case. While significant advancements have been made with these drugs, issues of drug resistance persist. To mitigate the impact of drug resistance and enhance patient survival rates, combination treatment strategies using two or three drugs are often employed to improve therapeutic effectiveness and prolong survival (19, 20) (**Table 1**).

**Table 1:** Clinical trials of some anticancer drugs.

Therapeutic drugs	Drug types	Patient types	Clinical data	Trial results	References
Doxorubicin + pembrolizumab	Chemotherapy drugs + targeted therapy drugs	Metastatic triple-negative breast cancer	Phase I NCT02648477	Some patients achieved good clinical remission, with 67% reaching the best remission and a 6-month clinical benefit rate of 56%.	(21)
Capivasertib	Targeted therapy drugs	Hormone receptor-positive advanced breast cancer	Phase III NCT04305496	In patients with advanced hormone receptor-positive breast cancer, treatment with capivasertib plus fulvestrant can significantly prolong progression-free survival (PFS).	(22)
Tamoxifen	Hormone therapy drug	Premenopausal patients with estrogen receptor-positive breast cancer	Phase III NCT00912548	For those who remain premenopausal or regain ovarian function, adding 2 years of ovarian function suppression (OFS) to tamoxifen (TAM) treatment can significantly improve disease progression-free survival (DFS) and overall survival.	(23)
Dalpiciclib plus letrozole or anastrozole	Hormone therapy drugs	Hormone receptor-positive, human epidermal growth factor receptor 2 (HER2)-negative advanced breast cancer	Phase III	Adding dalpiciclib to the therapy with letrozole or anastrozole significantly prolongs the patient’s disease progression-free survival (DFS). The treatment group receiving dalpiciclib showed notable results in extending disease-free time, although they experienced more grade 3 or 4 adverse events, mainly neutropenia and leukopenia.	(24)
Pertuzumab	Targeted therapy drugs	HER2-positive early breast cancer	Phase III NCT02586025	In patients treated with neoadjuvant pertuzumab plus trastuzumab and docetaxel, five-year event-free survival and disease-free survival rates were significantly better than those of the placebo group. The safety data indicated that the safety profile of the pertuzumab treatment group aligned with known expectations, with no significant differences between the groups, except for an increase in diarrhea.	(25)
Durvalumab + olaparib	Immunotherapy drugs + targeted therapy drugs	Triple-negative breast cancer	Phase I/II NCT02734004	The combination of olaparib and durvalumab demonstrated strong antitumor activity and safety that were comparable to those seen in monotherapy studies. Adverse events primarily included anemia, neutropenia, and pancreatitis, with no treatment-related deaths reported. Approximately 80% of patients achieved disease control at 12 weeks.	(26)
Trastuzumab	Targeted therapy drugs	HER2-positive metastatic breast cancer	Phase II	T-DXd exhibited sustained antitumor activity in these patients, evidenced by a high objective response rate of 62.0%. The safety profile was consistent, with most patients experiencing mild to moderate adverse events. However, there were a few severe adverse events, including drug-related interstitial lung disease and pneumonitis.	(27)
Pembrolizumab + carboplatin + docetaxel	Targeted therapy drugs + chemotherapy drugs	Triple-negative breast cancer	Phase II NCT03639948	This treatment regimen achieved a promising pathological complete response rate (pCR) and a three-year event-free survival in patients with triple-negative breast cancer. The regimen was well tolerated, and immune enrichment from various biomarker identifications independently predicted pCR.	(28)
Lapatinib	Targeted therapy drugs	HER2-negative metastatic breast cancer and HER2-positive circulating tumor cells	Phase III NCT01619111	Clearance of circulating tumor cells (CTCs) at the first follow-up was linked to improved overall survival. Furthermore, patients who received additional lapatinib treatment experienced a significant increase in overall survival.	(29)

## 3 Nanomaterials in breast cancer treatment

Nanomaterials can be classified based on various criteria. Common classifications include zero-dimensional (0D), one-dimensional (1D) nanomaterials, and polymers (30), as determined by their dimensions. Furthermore, when classified by chemical composition, nanomaterials can be divided into organic and inorganic categories (31). They may also be categorized according to their origin, material properties, applications, and morphology (32). Organic nanomaterials typically consist of substances found naturally in organisms or synthesized chemically. Compared to inorganic materials, they exhibit lower cytotoxicity and are biodegradable, making them a focal point of research in the field of nanomedicine (33). Inorganic nanomaterials are increasingly recognized as innovative tools for treating breast cancer due to their controlled drug release, multifunctionality, and excellent biocompatibility (18, 19, 34). The mechanisms by which nanomaterials assist in breast cancer treatment will be discussed in the following chapters. Nanomaterials used in breast cancer treatment are often categorized according to their chemical composition, as shown in **Table 2**.

**Table 2:** Advantages and disadvantages of several nanomaterials.

Nanomaterials	Advantages	Disadvantages	References
Solid lipid nanoparticles	Enhanced biopharmaceutical performance	Low encapsulation efficiency	(35)
Liposomes	Good biocompatibility		(36)
Polymeric nanoparticles	Multifunctional delivery	Prone to easy aggregation and toxicity	(37)
Magnetic nanomaterials	Controllable sustained release	Toxicity and solubility limitations	(38)
Magnetic nanomaterials	Stability and very high encapsulation efficiency		(39)
Quantum dots	Tunable optical properties, a large surface-to-volume ratio, high brightness, and resistance to photobleaching		(40, 41)

### 3.1 Solid lipid nanoparticles

**Figure 1:**
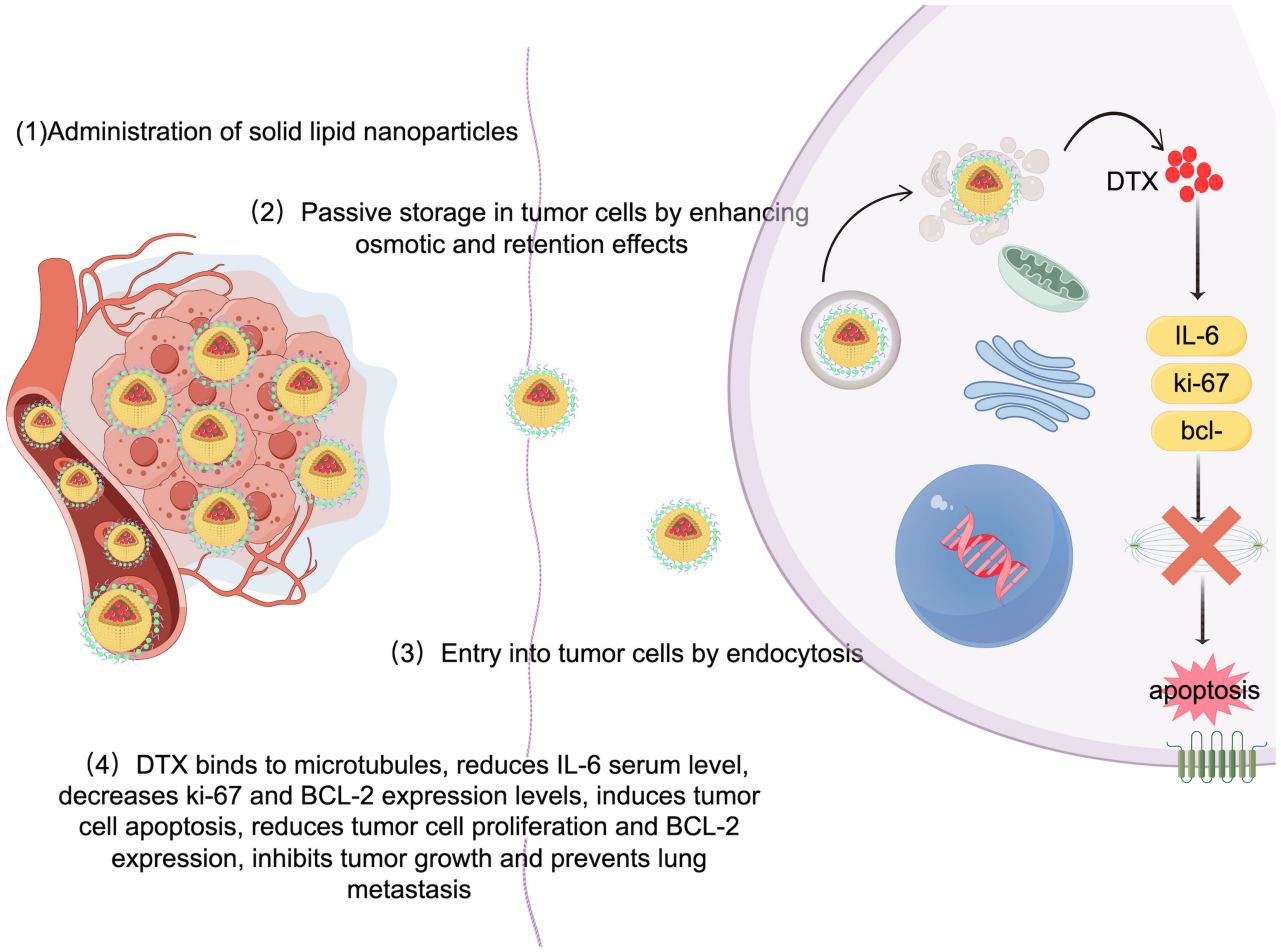
Mechanism of SLN-DTX in the treatment of breast cancer. Legend description: mechanism of SLN-DTX in the treatment of breast cancer (Created with Figdraw).

**Figure 2:**
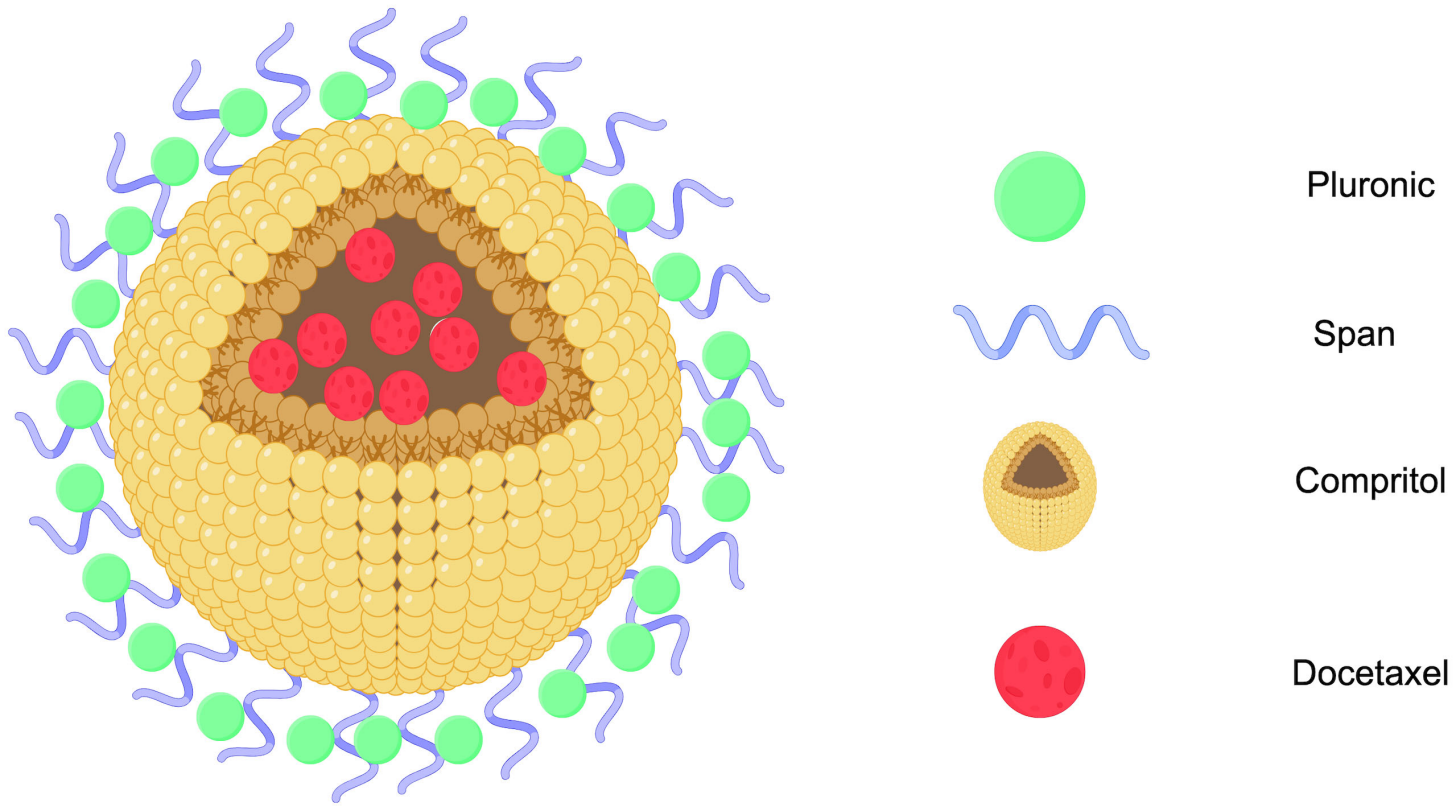
SLN-DTX structure diagram (Created with Figdraw). STN-DTX is assembled from four substances Pluronic, span, compritol and Docetaxel. The nanoparticles enhance drug uptake within breast cancer cells.

Solid lipid nanoparticles (SLNs) are made from safe physiological lipid components and exhibit good biocompatibility (42, 43). Granja et al. developed SLNs that encapsulate the antitumor drug mitoxantrone and functionalized them with 1,2-distearoyl-sn-glycero-3-phosphoethanolamine-N-[folate(polyethylene glycol) (DSPE-PEG-FA) ligands to enhance blood circulation and tumor selectivity. This reduces systemic side effects and enhances cellular uptake through processes such as macropinocytosis and clathrin-coated pits (43). CS/Lf/PTS-SLNs, prepared by Aly et al., improved drug solubility and bioavailability, achieving more effective tumor cell treatment. They enhance drug efficacy by inhibiting vascular endothelial growth factor, downregulating cyclin D1, and upregulating caspase-3 and BAX (44). SLN-DTX developed by da Rocha et al. enhances drug uptake in cells, promotes cell accumulation in the G2-M phase, and induces cell apoptosis, thereby increasing cytotoxicity. **Figure 1** illustrates the mechanisms by which SLN-DTX treatment induced tumor cell apoptosis, reduced tumor cell proliferation and BCL-2 expression, inhibited tumor growth, and prevented lung metastasis (45). **Figure 2** illustrates the four components of SLN-DIX: Pluronic, span, compritol and Docetaxel. Pindiprolu et al. demonstrated that PBA-Niclo-SLN induces G0/G1 cell cycle arrest and apoptosis, curtails STAT3, CD44/CD24 triple-negative breast cancer (TNBC) stem cell subsets, and markers of epithelial-mesenchymal transition, ultimately inducing tumor cell apoptosis and curtailing recurrence of TNBC (46). These findings indicate that solid lipid nanomaterials may serve as promising carriers for treating breast cancer and preventing its metastasis and recurrence.

### 3.2 Liposomes

**Figure 3:**
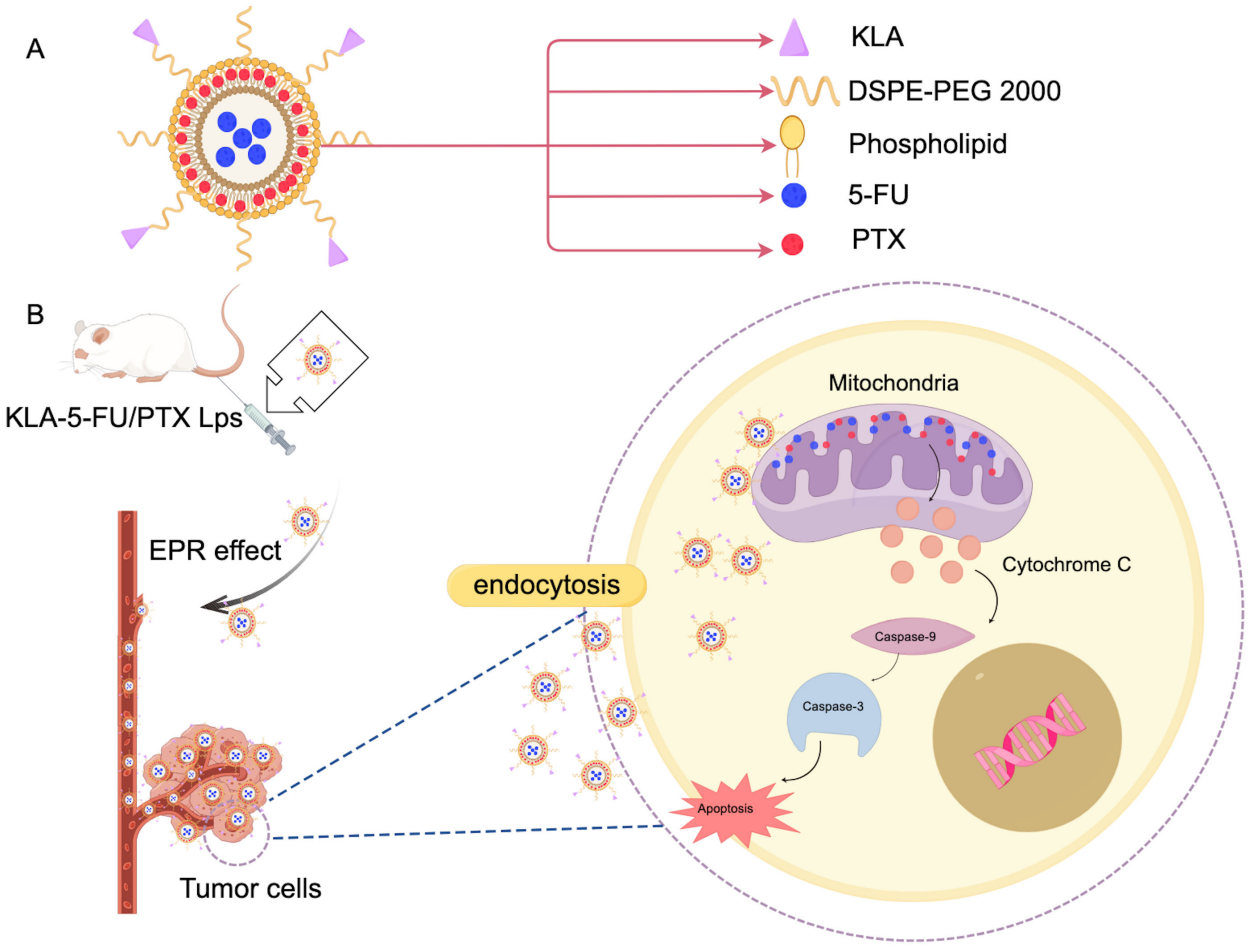
Mechanism of action of KLA in breast cancer treatment. **(A)** KLA and the main components of the administered liposomes. **(B)** This diagram illustrates the KLA peptide, which is delivered to the tumor cells through the EPR effect into the blood vessels. Mechanisms that promote tumor cells to endocytose liposomes, take up and selectively target mitochondria, thereby disrupting mitochondrial membranes. This process results in the loss of mitochondrial membrane potential, which in turn triggers the release of cytochrome C. The release of cytochrome C upregulates caspase-3 activity, thereby activating the apoptotic pathway in tumor cells. This mechanism provides a novel therapeutic strategy for the treatment of breast cancer and may have potential clinical applications. (Created with Figdraw).

Liposomes are nanocarriers characterized by a closed spherical vesicle structure made up of a phospholipid bilayer. With a composition similar to that of biological membranes, they can fuse with cell membranes to facilitate targeted intracellular drug release. Liposomes are capable of encapsulating both hydrophilic and hydrophobic drugs, enhancing drug loading, and providing sustained and controlled drug release (47). Boratto et al. highlighted the advantages of liposomes for intracellular delivery and release, positioning them as promising carriers for breast cancer treatment. Their pH-sensitive liposome preparation (pHSL-TS-DOX) demonstrated improved drug delivery at the intracellular level, greater accumulation in tumors, and sustained release of doxorubicin (DOX). Additionally, treatment with pHSL-TS-DOX resulted in cell cycle arrest primarily in the G1 phase, which may contribute to slowing the proliferation of tumor cells (48). Furthermore, APA-functionalized liposomes (EGA-EML-APA) developed by Badr-Eldin et al. enhance cytotoxicity against human breast cancer cells. By inducing G2/M and S phase arrest in MCF-7 cells, this process includes upregulating the expression of p53, bax, and casp3, downregulating bcl2, reducing NF-κB activity, increasing TNFα expression, and triggering significant apoptotic activity (49). Chen et al. created KLA-modified liposomes co-loaded with 5-fluorouracil and paclitaxel (KLA-5-FU/PTX Lps), which displayed enhanced cytotoxicity against MDA-MB-231 cells. The proposed anti-tumor mechanism in **Figure 3** suggests that the KLA peptide facilitates liposome absorption into tumor cells and selectively targets mitochondria, leading to mitochondrial membrane disruption, the loss of membrane potential, cytochrome C release, caspase-3 upregulation, and activation of the apoptotic pathway in tumors (**Figure 3**). As such, KLA-5-FU/PTX Lps is a promising system for treating TNBC (50). Collectively, these studies indicate that functionalized liposomes hold significant potential in the fight against breast cancer.

### 3.3 Polymer nanomaterials

Polymers possess a distinctive “core-shell” structure made up of micelles formed from amphiphilic molecules. In recent years, polymeric nanoparticles have emerged as significant candidate carriers in drug delivery due to their advantages, including biodegradability, improved encapsulation efficiency, biocompatibility, and controlled release (51, 52). Furthermore, by modifying the surface with ligands or targeting agents, polymers can enable multifunctional drug delivery. Bressler et al. demonstrated that by attaching AXT050 to polylactic acid-co-polylactic acid (PLGA)-PEG nanoparticles, they could accurately control the surface density of the nanoparticles. This modification allows them to serve as targeting agents for human tumor cells while exhibiting anti-tumor and anti-angiogenic properties. Such targeted modifications enhance the effectiveness of cancer nanomedicines and improve their ability to combat tumor growth (53). A study by Sharma et al. indicated that nanoparticles could effectively deliver curcumin into MDA-MB231 breast cancer cells, significantly enhancing both encapsulation efficiency and drug release capabilities. These nanoparticles have also shown the ability to diminish cell viability and suppress cell invasion and metastasis (54). Barkat et al. utilized PNP-loaded polymer nanoformulations that may aid in breast cancer treatment by restoring mitochondrial function and decreasing reactive oxygen species production and intracellular ATP levels (55). Polymer nanoparticles are characterized by their biodegradability, efficient encapsulation, controlled release, and biocompatibility, showcasing promising applications in the field of breast cancer treatment.

### 3.4 Magnetic nanoparticles

Magnetic nanoparticles are known for their excellent biocompatibility, controllable sustained release, and targeting capabilities, as well as their extremely high magnetocaloric effect. For example, chitosan-coated iron-manganese oxide nanoparticles (CS-MIONP) effectively conduct heat, making them an area of great interest in the field of hyperthermia for tumor cell destruction (56). Li et al. designed MUC1-C shRNA@Fe3O4MNPs, which can rapidly and stably generate heat under a constant alternating magnetic field at specific concentrations. This provides strong technical support for hyperthermia therapy in cancer. Through endocytosis, TNBC cells can effectively absorb these nanoparticles, significantly dampening their proliferation and migration. After treatment, the expression levels of key proteins in both TNBC cells and tumor tissues were significantly reduced, while the levels of apoptosis-related proteins were notably increased. This finding not only reveals the mechanism by which MUC1-C shRNA@Fe3O4MNPs impede tumor growth but also offers an important theoretical foundation for developing future cancer treatment strategies (57). As an advanced nanocarrier system, magnetic nanoparticles (TMX-AG-INP) can efficiently deliver various chemotherapy drugs, such as methotrexate and doxorubicin, to breast cancer tissues. They can also bind to gold nanoparticles and bovine albumin, producing potent anti-tumor effects. These INPs can accurately deliver miR-29a (micro-RNA) to breast cancer tissue after being coated with dextran, effectively diminishing the expression of anti-apoptotic genes. This represents a new strategy for breast cancer treatment (58). The combination of drugs with magnetic nanosystems has shown great potential for targeted drug delivery (59). This approach not only optimizes drug dosage to minimize side effects but also significantly enhances the cytotoxic effect on cancer cells, offering more effective, precise, and safer prospects for cancer treatment (60, 61).

### 3.5 Quantum dots

Quantum dots are semiconductor nanocrystals with sizes ranging from 2 to 10 nm and are versatile tools in biological imaging, diagnosis, and combination therapy due to their fluorescence properties (62, 63). However, one limitation of these quantum dots is their high hydrophobicity, necessitating surface coatings with polymers or multilayer ligand shells to enhance solubility. Typically, these quantum dots feature a semiconductor metal core composed of elements from groups II to VI or III to V, and their physical and chemical properties can be modified through the addition of surface ligands or polypeptides, which is particularly useful in targeted cancer treatments (64). Karami et al. developed an innovative water-in-oil-in-water double nanoemulsion method to create hydrogel nanocarriers incorporating chitosan and alumina (γ-Al2O3) along with carbon quantum dots. This method effectively addresses the challenges of low solubility and short half-life of drugs such as curcumin, significantly enhancing their effectiveness in drug release systems (65). Notably, the drug loading capacity and encapsulation efficiency of this nanocarrier surpass those of previously reported curcumin nanocarriers. Additionally, graphene quantum dots, prepared by another research team (66), exhibit low toxicity. Following exposure to cancer cells, there is a corresponding increase in the expression levels of p21 and p27. Most impressively, cells treated with these orthogonal graphene quantum dots demonstrated G2/M phase arrest and specifically induced apoptosis in estrogen receptor-positive breast cancer cell lines, opening new avenues for cancer treatment and research.

## 4 Mechanism of action of nanomaterials in alleviating drug resistance in breast cancer

**Figure 4:**
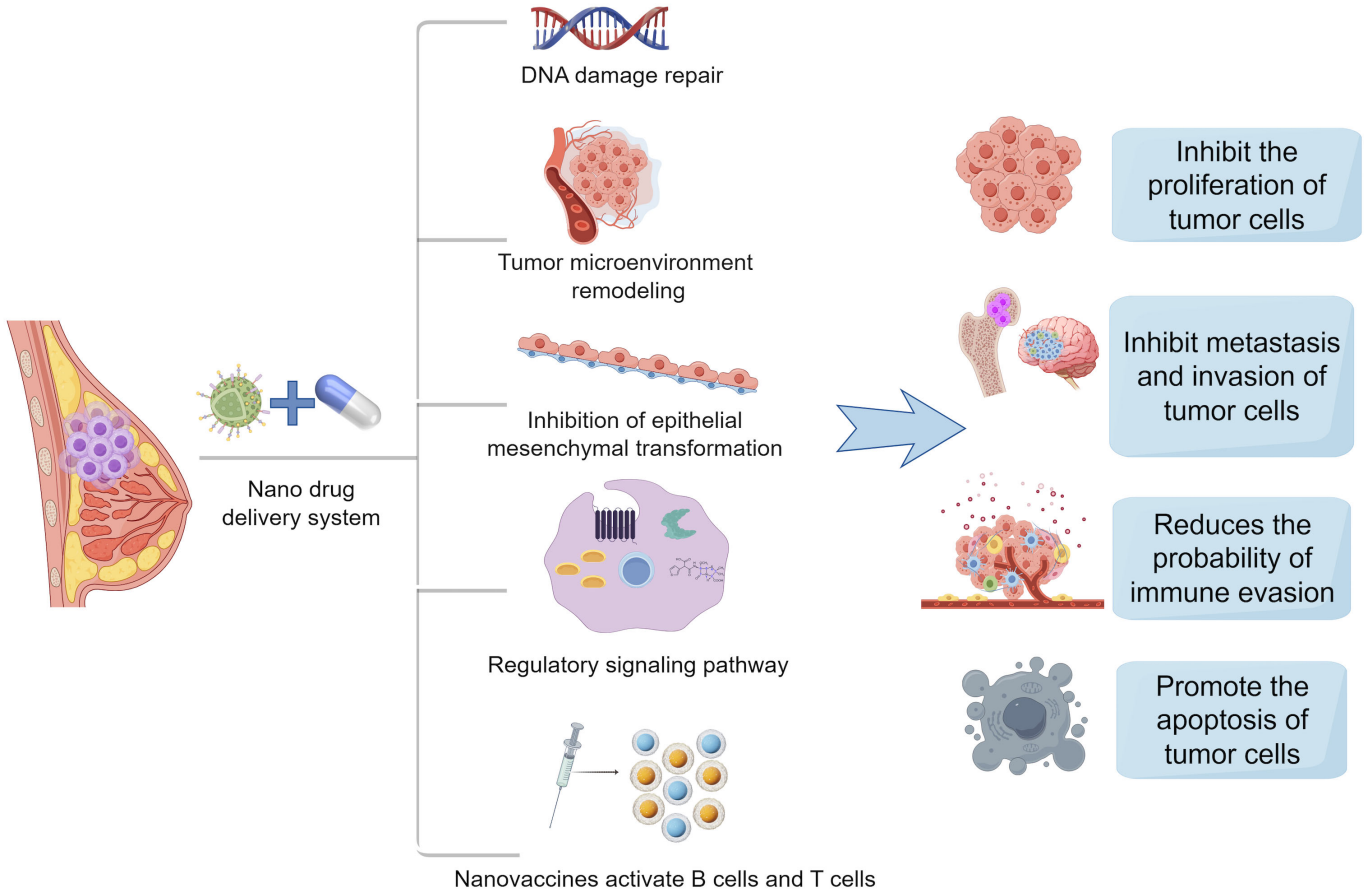
Mechanisms of action of nanodrug delivery systems in breast cancer therapy. Nanodrug delivery systems in the treatment of breast cancer often inhibit the proliferation of tumor cells, inhibit the metastasis and invasion of tumor cells, reduce immune escape, and promote the apoptosis of tumor cells by DNA damage repair, remodeling of tumor microenvironment, inhibition of epithelial mesenchymal transition, regulation of signaling pathways, and activation of B cells and T cells by nanovaccines.(Created with Figdraw).

Chemotherapy is one of the most common treatments for aggressive breast cancer, particularly TNBC. To tackle the challenge of drug resistance in breast cancer, various strategies can be employed, including blocking drug efflux and inactivation (8), inhibiting alternative signaling pathways, addressing DNA repair (67), suppressing the EMT process, and diminishing cancer stem cell characteristics. Nanomaterials are engaged in dampening tumor cell proliferation (68), metastasis, and invasion (69), as well as delivering chemotherapeutic drugs, promoting angiogenesis, evading immune responses, and remodeling the tumor microenvironment. Numerous studies have demonstrated that nanomaterials play a crucial role in overcoming chemotherapy resistance, the potential therapeutic mechanism of BC resistance are summarized in **Figure 4**, which offering promising therapeutic prospects for breast cancer treatment (70–72).

### 4.1 Inhibition of drug efflux

**Figure 5:**
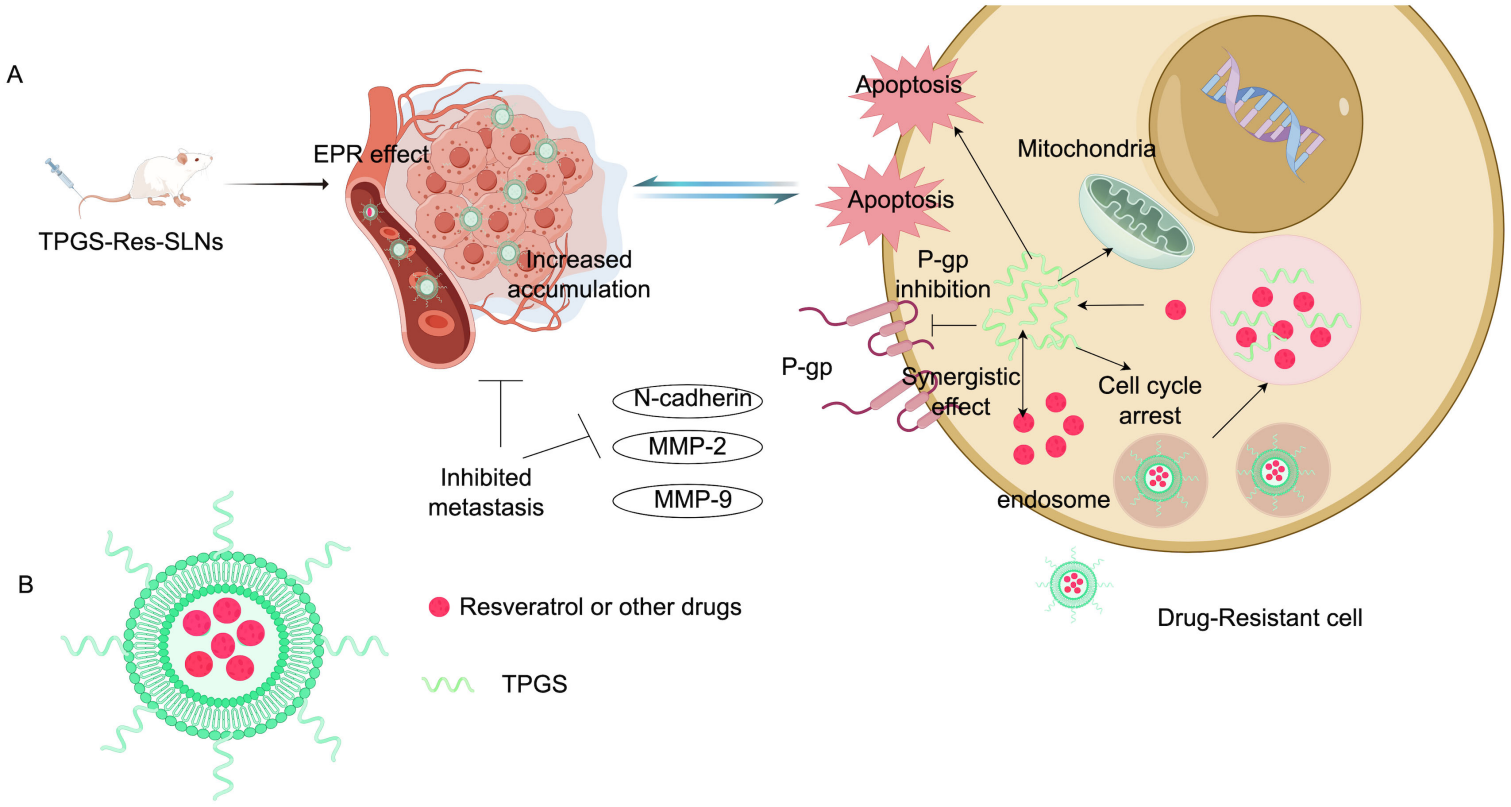
Composition and mechanism of TPGS-Res-SLNs. **(A)** Illustration: TPGS-Res-SLNs reduce the expression of P-gp and BCRP-related proteins. TPGS-Res-SLNs also refrain from the epithelial-mesenchymal transition of cancer cells and attenuate the invasion and metastasis of cancer cells. **(B)** Characterization diagram of TPGS-Res-SLNs (Created with Figdraw).

Cytotoxic drugs need to be fully taken up by cancer cells to effectively treat cancer. Increasing drug uptake may help reduce chemotherapy resistance (73, 74). One of the most common mechanisms behind chemotherapy resistance is the drug efflux mediated by transmembrane pumps. To combat this issue, researchers have utilized acid-grafted poly(β-amino ester) nanoparticles to encapsulate cleavage protein B, aiming to overcome multidrug resistance to chemotherapy drugs. These nanoparticles have a pH-sensitive release function, which enhances drug release as the pH decreases. Importantly, they can also reverse multidrug resistance by halting the expression of P-glycoprotein (P-gp) and affecting the energy supply for drug efflux, thereby providing a new strategy for breast cancer treatment (75). Guo’s team successfully developed folic acid-modified nanoparticles ((DOX + CUR)-FA-NPs) based on star-shaped polyester (FA-TRI-CL), which are particularly effective in curbing P-gp to counteract resistance. P-gp is a transmembrane transporter that expels drugs from cells, leading to chemotherapy resistance. By impeding P-gp, (DOX + CUR)-FA-NPs significantly prevent resistant cells from excreting the drugs, thus enhancing their accumulation in tumor cells and alleviating chemotherapy resistance (76). Researchers also prepared D-α-tocopherol polyethylene glycol 1000 succinate-resveratrol solid lipid nanoparticles (TPGS-Res-SLNs), which successfully reduced the expression of multidrug resistance-related proteins such as P-gp and BCRP. This reduction diminishes the excretion and inactivation of chemotherapy drugs in cells, increasing both the concentration and duration of drug action. Furthermore, TPGS-Res-SLNs curtailed the EMT of cancer cells, decreasing their ability to invade and metastasize, while enhancing the effectiveness of chemotherapy drugs. In summary, TPGS-Res-SLNs represent a promising approach to alleviate drug resistance in breast cancer (77). **Figure 5** shows the composition and mechanism of tpgs-res-sln (**Figure 5**). Cancer cells develop drug resistance partly by using efflux pumps (such as P-gp and BCRP) to expel anticancer drugs, which lowers drug concentrations in cells and weakens treatment effects. Ptx-SLN bypasses these efflux pumps in breast cancer cells, facilitating better drug entry and improving anticancer effects, especially in multidrug-resistant cells, offering new hope for treating drug-resistant breast cancer (78). Recent studies have introduced an intelligent and multifunctional MoS2 nano-theranostic platform (MoS2-PEI-HA) that targets CD44-overexpressing cancer cells and decomposes in the tumor microenvironment due to hyaluronidase, which accelerates the release of the chemotherapeutic drug DOX. The MoS2 also enhances near-infrared photothermal conversion, promoting DOX release in the acidic environment of tumors through mild laser irradiation (74). Most notably, this technology can downregulate the expression of P-gp, which is associated with drug resistance, thereby reducing drug efflux, increasing intracellular drug accumulation, and effectively reversing drug resistance. Guney et al. developed a new type of talazoparib SLN and found that the efflux of talazoparib in TNBC cells was mediated by BCRP and MRP1 pumps. Talazoparib-SLNs significantly improved the therapeutic effects of talazoparib and suppressed the expression of MDR1, BCRP, and MRP1 genes and proteins. In summary, targeting the expression of genes related to drug efflux pumps is an effective strategy for alleviating drug resistance in breast cancer (79).

### 4.2 Interference with signaling pathways

Cell signaling plays a crucial role in the key stages of hormone-independent breast cancer tumorigenesis, including cell proliferation, survival, angiogenesis, and metastasis (80). Understanding the abnormalities in the signaling pathways that contribute to resistant breast cancer cells can help identify specific peptides and modify their properties to selectively target these pathways. This section provides a brief overview of the role these signaling pathways play in breast cancer chemotherapy resistance. Defects in the PI3K/AKT/mTOR signaling pathways are known to cause multidrug resistance and promote cancer metastasis. Yin et al. developed a pH-sensitive nanocomplex that co-delivers paclitaxel (PTX) and siRNA to metastatic breast cancer. The siRNA targets and silences Akt expression in 4T1 metastatic breast cancer cells. In these cells, PTX-loaded micelle/siAkt nanocomplexes (PMA) successfully downregulated P-gp, upregulated Caspase-3 expression, and resulted in Akt gene knockdown. Beyond promising *in vitro* results, PMA demonstrated both efficacy and safety *in vivo*. In 4T1 tumor-bearing mice, PMA achieved 94.1% tumor inhibition and 96.8% reduction in breast cancer lung metastasis. Furthermore, the PMA nanocomplex is characterized by very low toxicity and does not cause lesions in normal tissues (81). Guney et al. discovered that TNBC cells often overexpress Notch1 receptors while underexpressing miR-34a microRNA. To tackle this issue, they encapsulated miR-34a mimics within poly(lactic-co-glycolic acid) nanoparticles (NPs), creating N1-34a-NPs. These nanoparticles were functionalized to bind to the overexpressed Notch1 receptors on the surface of TNBC cells, thereby interfering with Notch signaling through the associated signaling cascade. Their experiments showed that N1-34a-NPs could effectively regulate Notch signaling and its downstream targets in TNBC cells, leading to cell senescence and reduced proliferation and migration. This study suggests that nanoparticle-mediated co-delivery of miR-34a and Notch1 antibodies may serve as a promising alternative treatment strategy for TNBC, warranting further optimization and *in vivo* investigation (79). Additionally, studies have indicated that Dox-HBDL nanoparticles significantly enhance therapeutic effects on drug-resistant breast cancer cells. This nanoparticle activates the intracellular JNK pathway, promoting apoptosis and inducing the dissipation of mitochondrial membrane potential while increasing reactive oxygen species levels in the cytoplasm. This mechanism further activates the pro-apoptotic JNK signaling in drug-resistant cells, hindering drug degradation and increasing apoptosis and cell death (82). Cheng et al. reported similar findings in nanoparticles that co-deliver DOX and pyrrolidine dithiocarbamate, where pyrrolidine dithiocarbamate served as a chemosensitizer, enhancing intracellular drug levels by halting the NF-κB pathway (83). The researchers designed pH-sensitive nanoparticles from poly(orthoester carbamate) copolymers, which are stable in neutral pH environments but degrade rapidly in slightly acidic conditions (84, 85). Liang et al. identified novel functional roles for heat shock protein beta-1 (HSPB1) in regulating chemoresistance and ferroptotic cell death in breast cancer. HSPB1 could bind with Ikβ-α and promote its ubiquitination-mediated degradation, leading to increased nuclear translocation and activation of NF-κB signaling. Thus, targeting HSPB1 and combinational induction of ferroptosis with anticancer drugs would be a potential therapeutic strategy to overcome chemoresistance in breast cancer (86).

### 4.3 Inhibition of epithelial-mesenchymal transition

A recent study demonstrates that present a novel approach of combinatorial treatment of DOX with dietary indole 3,3’-diindolylmethane (DIM). They combined DIM with DOX and delivered to the CSCs concomitantly by loading them in mesoporous silica nanoparticles encapsulated in exosomes (e-DDMSNP), which improved the specificity, stability and better homing ability of DIM and DOX in the *in vitro* and *in vivo* CSC niche. Therefore, this novel exosome nanopreparation has great potential to target CSC and EMT (87).SKN@FPD NM is a novel type of nanodrug delivery system. By leveraging the inhibitory effects of SKN, this nanomaterial can effectively disrupt the epithelial-mesenchymal transition process, thereby reducing the metastatic potential of breast cancer cells. Furthermore, this combination therapy enhances tumor cell toxicity while reducing the risk of drug resistance and treatment failure (88). In summary, nanodrug delivery systems offer a promising strategy for overcoming drug resistance in breast cancer.

Nanodrug-carrying systems have also demonstrated remarkable effectiveness in addressing drug resistance across various malignant cancers. For instance, some researchers have developed a nanosystem based on programmed DNA self-assembly of Rab26 siRNA-loaded nanoparticles for treating drug resistance in lung cancer (66). Additionally, miRNA and PTX can be delivered through RAW-PANP, presenting a new method for tackling PTX-resistant TNBC. Simultaneously, RAW-PANP can serve as an effective drug delivery system for targeted therapy in TNBC (89).

## 5 Application of targeted nano-drug delivery in breast cancer drug resistance

### 5.1 Passive drug targeting

In normal tissues, the vascular structure is compact, whereas in tumor sites, it is abnormal with wider pores. This difference allows nanomaterials to penetrate tumor tissue more easily, a phenomenon known as the enhanced permeability retention effect (90). Passive targeting is a drug delivery approach that leverages these anatomical and physiological differences in tissue. Specifically, it utilizes the enhanced permeability retention effect, which is influenced by certain changes in tumor tissues, such as large gaps in the blood vessel endothelium, limited lymphatic drainage, and disrupted tissue architecture. These factors increase the likelihood of macromolecular particles gathering in tumor sites (91). Nanomaterials can further enhance the enhanced permeability retention effect by adjusting their particle size at the tumor area, thereby increasing their effectiveness against breast cancer (35, 92). These nanomaterials can be categorized into various delivery methods, including liposomes, micelles, nanogels, and nanoparticles (93). Addressing common challenges in treating TNBC, such as chemotherapy resistance and off-target damage, researchers led by Chen et al. developed a novel type of nanoparticle. This formulation combines polylactic acid-co-polylactic acid with a lipid shell, enabling it to carry drugs such as paclitaxel and anti-miR-221, along with perfluoropropane for ultrasound-triggered release. These nanoparticles are delivered to the tumor site by macrophages, utilizing the innate targeting ability of these cells to enhance treatment precision. Experimental results demonstrate that this system significantly increases the sensitivity of TNBC cells to chemotherapeutic agents while minimizing harm to normal tissues (94). Additionally, Xiong et al. created DOX-TA-ICG particles (DTIG) with a diameter of (74 ± 2) nm. Upon arrival at the tumor site, DTIG experiences increased proton concentration, causing them to further condense through electrostatic interactions, which enhances their uptake by tumor cells. As proton concentration rises in the lysosome (pH = 4.5), DTIG aggregation becomes more pronounced, ultimately forming larger particles with a diameter of 1.5 μm that escape the lysosome. Once in the cytoplasm, the near-neutral environment prompts DTIG to quickly transform into larger hydrophilic particles measuring 120-200 nm (95). In summary, at the tumor site, the high permeability and retention characteristics of nanomaterials may improve the accumulation of chemotherapeutic drugs, thereby mitigating breast cancer resistance and reducing off-target damage (93).

### 5.2 Active drug targeting

Targeted molecular therapy enhances the precision of cancer treatments and is one of the most effective methods in targeted therapy. Active targeting relies on the interaction between a targeting agent and its corresponding receptor, allowing nanoparticles to specifically bind to antigens expressed by tumor cells. This method improves drug delivery efficiency and specificity (96). In breast cancer treatment, several key molecular targets have been identified, including human epidermal growth factor receptor 2, vascular endothelial growth factor, folate receptors, somatostatin receptors, vascular endothelial cell adhesion molecules, estrogen receptors, and cyclooxygenase-2 (97). A study by Mamnoon et al. developed hypoxia-responsive polymer nanoparticles (HRPs) functionalized with 17β-estradiol (E2) for the targeted delivery of DOX in estrogen receptor-positive breast cancer cells. They prepared estradiol-coupled polymer-DOX complexes (E2-DOX-HRPs), which demonstrated a higher DOX loading efficiency compared to non-targeted preparations. Under hypoxic conditions, E2-DOX-HRPs exhibited enhanced antiproliferative effects, significantly reducing cell viability and causing spheroid shrinkage in three-dimensional cultures of MCF-7 cells. These findings indicate that the targeted HRP formulation successfully recognized estrogen receptors on breast cancer cells and released more DOX under tumor hypoxia, enhancing the anticancer effect of DOX and effectively alleviating chemotherapy resistance (98).

### 5.3 The physical and chemical targeting effect of nanocarriers enhances the targeting of breast cancer cells

Physical and chemical targeting is a method to enhance the targeted therapeutic effects on cancers such as breast cancer by precisely delivering drugs to specific transport sites through external forces such as temperature, magnetic fields, and pH. Temperature-sensitive carriers can be made into thermosensitive agents, allowing them to release drugs in thermotherapy target areas (99); or magnetic materials can be applied to form magnetic guidance agents with drugs, which, guided by external magnetic fields *in vitro*, reach and localize in specific target areas through the bloodstream (100); pH-sensitive carriers can also be utilized to prepare pH-sensitive agents, enabling drug release in specific target areas (101). Embolic agents can block blood supply and nutrition in target areas, serving a dual role in embolization and targeted chemotherapy. Puluhulawa et al. used Chemical grafting of chitosan nanoparticles with hyaluronic acid as a targeted ligand to control drug release through ph-responsive stimulation, and the high selectivity of hyaluronic acid for CD44 receptors allowed these nanoparticles to accumulate more in cells that overexpress these receptors (102). Zhang et al. developed the “cell targeting destructive” multifunctional polymeric nanoparticles (HA-Olb-PPMNPs). Under rotating magnetic field (RMF), HA-Olb-PPMNPs can produce physical transfer of mechanical force through incomplete rotation, which can cause a “two-strike” effect on cells, in which the “first strike” is to destroy the membrane structure, and the other “second strike” is to activate the lysosomal-mitochondrial pathway to induce apoptosis by damaging the lysosome (103). Nasri et al. developed a new monoclonal antibody conjugated dual stimuli lipid-coated mesoporous silica nanoparticles (L-MSNs) platform, first synthesizing MSN and then using prepared pH and heat-sensitive niosome coated to produce L-MSN. Use a key-lock interaction between trastuzumab and HER-2 receptors on the cancer cell membrane to deliver specifically targeted drugs to the cancer cell, stimulating the endocytosis of particles into the cell, which then disrupts the lipid layer at acidic pH and lysosome temperature, leading to enhanced release of PTX and GEM (104).

## 6 Research progress in anticancer nanomaterials

Chemotherapy, surgery, hormone therapy, radiotherapy, and targeted therapy form the core strategies for breast cancer treatment. Despite these methods, researchers continue to explore new treatments and drugs. Patients participating in clinical trials not only have the opportunity to try potentially advanced therapies but also contribute to cancer research, fostering collaboration among breast cancer patients. The U.S. Food and Drug Administration has officially approved the use of palbociclib, ribociclib, and everolimus in combination with hormone therapy for the treatment of advanced or metastatic breast cancer. Clinical studies have shown that ribociclib can effectively extend the survival of patients with metastatic breast cancer. Additionally, Abemaciclib is approved for use alongside or following hormone therapy, specifically for patients with hormone receptor-positive, human epidermal growth factor receptor 2-negative advanced or metastatic breast cancer. **Table 3** provides a list of drugs approved by the U.S. Food and Drug Administration from 2021 to 2023.

**Table 3:** List of the drugs approved by U.S. Food and Drug Administration from 2021–2023 for advanced or metastatic breast cancer.

Drug molecule	Brand name/Manufacturer	Approval date	Remarks	References
Capivasertib	Truqap/AstraZeneca Pharmaceuticals LP	2023/11/16	Capivasertib is a novel, selective ATP-competitive pan-AKT kinase inhibitor used in combination with fulvestrant to treat adult patients with hormone receptor (HR)-positive, human epidermal growth factor receptor 2 (HER2)-negative, locally advanced or metastatic breast cancer.	(fda.gova)
Elacestrant	Orserdu/Stemline Therapeutics, Inc.	2023/01/27	Elacestrant is an oral selective estrogen receptor degrader that treats postmenopausal women or adult men with ER-positive, HER2-negative, estrogen receptor 1 (ESR1) mutation-≥advanced or metastatic breast cancer with disease progression after first-line endocrine therapy.	(fda.gova)
Fam-trastuzumab deruxtecan-nxki	Enhertu/Daiichi Sankyo, Inc	2022/08/05	Fam-trastuzumab deruxtecan-nxki is a HER2 antibody drug conjugate combined with irinotecan-type chemotherapy drug, which belongs to the ADC-type drug. It is used to treat adult patients with unresectable or metastatic HER2-positive breast cancer who have previously received two or more anti-HER2-based regimens.	(fda.gova)
Olaparib	Lynparza/AstraZeneca Pharmaceuticals, LP	2022/03/11	Olaparib is an oral PARP inhibitor designed for patients with HER2-negative advanced breast cancer who carry germinal BRCA1/2 mutations.	(fda.gova)
Abemaciclib	Verzenio/Eli Lilly and Co.	2021/10/12	Abemaciclib is an oral, continuously administered CDK4/6 inhibitor used for treating patients with advanced breast cancer who are HR-positive and HER2-negative.	(fda.gova)
Pembrolizumab	Keytruda/Merck	2021/07/26	Pembrolizumab is a humanized monoclonal anti-PD1 antibody approved for treating high-risk, early-stage triple-negative breast cancer (TNBC) patients.	(fda.gova)

## 7 Challenges and hopes

Cancer drug resistance remains a significant challenge in current cancer treatments and is responsible for the majority of cancer-related fatalities. However, the versatility of nanomaterials offers a revolutionary approach to combat this issue and provides new hope. These materials can serve both diagnostic and therapeutic functions, allowing for precise tumor localization and drug delivery while enhancing treatment efficacy and minimizing harm to healthy tissues. Advancements in this technology are expected to lead to breakthroughs in cancer therapy and offer patients more personalized and effective treatment options. This review begins by classifying different nanomaterials and explaining their mechanisms of action, particularly in breast cancer research. Nonetheless, the design, optimization, and potential biomedical applications of these nanomaterials require further investigation.

Firstly, nanomaterials’ capability to deliver functional substances to diseased cells makes them promising therapeutic carriers and potential targets for overcoming drug resistance. However, preparing nanomaterials to target specific sites demands significant time, effort, and financial resources, complicating their clinical application. Furthermore, identifying the optimal target carriers to develop the most effective combined strategies while minimizing adverse effects in treatment should be a key focus for future research in this area. Secondly, while nanomaterials have demonstrated good tolerance and minimal side effects, their toxicity remains a critical concern in medical and environmental applications. Additionally, nanomaterials can facilitate dynamic measurement of various biological components associated with tumor resistance, holding unique potential for monitoring cancer’s complex dynamics and potentially serving as candidate biomarkers for predicting and assessing treatment outcomes in breast cancer patients. However, optimizing detection methods and maximizing the advantages of nanomaterials in breast cancer treatment to achieve synergistic effects of “1 + 1>2” still requires extensive research and exploration. Despite their many beneficial properties, nanomaterials can also present toxic effects due to their unique physical and chemical characteristics. For instance, their small size and high surface area may increase the likelihood of interactions with biological systems, leading to adverse effects such as cytotoxicity, immunotoxicity, and genotoxicity, which can result in inflammatory responses, oxidative stress, and cellular damage, potentially disrupting physiological functions. Thus, careful attention to toxicity issues, along with implementing safety measures during the design, synthesis, and application of nanomaterials, is essential to ensure their safety and reliability.

Despite the substantial challenges, with ongoing advancements in science and technology and the progress of clinical research, we believe that nanomaterials have the potential to become vital tools in treating breast cancer resistance and bring renewed hope to patients.
